# COVID-19 Vaccine Breakthrough Infections Reported to CDC — United States, January 1–April 30, 2021

**DOI:** 10.15585/mmwr.mm7021e3

**Published:** 2021-05-28

**Authors:** CDC COVID-19 Vaccine Breakthrough Case Investigations Team, CDC COVID-19 Vaccine Breakthrough Case Investigations Team, Meseret Birhane, Sara Bressler, Gregory Chang, Thomas Clark, Layne Dorough, Marc Fischer, Louise Francois Watkins, Jason M. Goldstein, Kiersten Kugeler, Gayle Langley, Kristin Lecy, Stacey Martin, Felicita Medalla, Kiren Mitruka, Leisha Nolen, Katrin Sadigh, Robin Spratling, Gail Thompson, Alma Trujillo

**Affiliations:** CDC; CDC; CDC; CDC; CDC; CDC; CDC; CDC; CDC; CDC; CDC; CDC; CDC; CDC; CDC; CDC; CDC; CDC; CDC.

COVID-19 vaccines are a critical tool for controlling the ongoing global pandemic. The Food and Drug Administration (FDA) has issued Emergency Use Authorizations for three COVID-19 vaccines for use in the United States.* In large, randomized-controlled trials, each vaccine was found to be safe and efficacious in preventing symptomatic, laboratory-confirmed COVID-19 (*1*–*3*). Despite the high level of vaccine efficacy, a small percentage of fully vaccinated persons (i.e. received all recommended doses of an FDA-authorized COVID-19 vaccine) will develop symptomatic or asymptomatic infections with SARS-CoV-2, the virus that causes COVID-19 (*2*–*8*).

CDC is working with state and territorial health departments to investigate SARS-CoV-2 infections among persons who are fully vaccinated and to monitor trends in case characteristics and SARS-CoV-2 variants identified from persons with these infections. For this surveillance, a vaccine breakthrough infection is defined as the detection of SARS-CoV-2 RNA or antigen in a respiratory specimen collected from a person ≥14 days after receipt of all recommended doses of an FDA-authorized COVID-19 vaccine. State health departments voluntarily report vaccine breakthrough infections to CDC.^†^ When possible, genomic sequencing is performed on respiratory specimens that test positive for SARS-CoV-2 RNA (*9*).

A total of 10,262 SARS-CoV-2 vaccine breakthrough infections had been reported from 46 U.S. states and territories as of April 30, 2021. Among these cases, 6,446 (63%) occurred in females, and the median patient age was 58 years (interquartile range = 40–74 years). Based on preliminary data, 2,725 (27%) vaccine breakthrough infections were asymptomatic, 995 (10%) patients were known to be hospitalized, and 160 (2%) patients died. Among the 995 hospitalized patients, 289 (29%) were asymptomatic or hospitalized for a reason unrelated to COVID-19. The median age of patients who died was 82 years (interquartile range = 71–89 years); 28 (18%) decedents were asymptomatic or died from a cause unrelated to COVID-19. Sequence data were available from 555 (5%) reported cases, 356 (64%) of which were identified as SARS-CoV-2 variants of concern,^§^ including B.1.1.7 (199; 56%), B.1.429 (88; 25%), B.1.427 (28; 8%), P.1 (28; 8%), and B.1.351 (13; 4%).

As of April 30, 2021, approximately 101 million persons in the United States had been fully vaccinated against COVID-19.^¶^ However, during the surveillance period, SARS-CoV-2 transmission continued at high levels in many parts of the country, with approximately 355,000 COVID-19 cases reported nationally during the week of April 24–30, 2021.** Even though FDA-authorized vaccines are highly effective, breakthrough cases are expected, especially before population immunity reaches sufficient levels to further decrease transmission. However, vaccine breakthrough infections occur in only a small fraction of all vaccinated persons and account for a small percentage of all COVID-19 cases (*5*–*8*). The number of COVID-19 cases, hospitalizations, and deaths that will be prevented among vaccinated persons will far exceed the number of vaccine breakthrough cases. To date, the age and sex distribution of reported vaccine breakthrough infections reflects the fully vaccinated U.S. population.^††^ The proportion of reported vaccine breakthrough infections attributed to variants of concern has also been similar to the proportion of these variants circulating throughout the United States. During March 28–April 10, 2021, the aforementioned variants of concern accounted for 70% of the weighted estimates of SARS-CoV-2 lineages submitted to CDC’s national genomic surveillance.^§§^

The findings in this report are subject to at least two limitations. First, the number of reported COVID-19 vaccine breakthrough cases is likely a substantial undercount of all SARS-CoV-2 infections among fully vaccinated persons. The national surveillance system relies on passive and voluntary reporting, and data might not be complete or representative. Many persons with vaccine breakthrough infections, especially those who are asymptomatic or who experience mild illness, might not seek testing. Second, SARS-CoV-2 sequence data are available for only a small proportion of the reported cases.

Beginning May 1, 2021, CDC transitioned from monitoring all reported COVID-19 vaccine breakthrough infections to investigating only those among patients who are hospitalized or die, thereby focusing on the cases of highest clinical and public health significance. CDC will continue to lead studies in multiple U.S. sites to evaluate vaccine effectiveness and collect information on all COVID-19 vaccine breakthrough infections regardless of clinical status. Additional information and resources to help public health departments and laboratories investigate and report COVID-19 vaccine breakthrough cases are available at https://www.cdc.gov/vaccines/covid-19/health-departments/breakthrough-cases.html.

FDA-authorized COVID-19 vaccines are safe and effective (*1*–*8*). CDC recommends that all persons aged ≥12 years be vaccinated with an FDA-authorized COVID-19 vaccine^¶¶^ (*10*).

## Footnotes

*
https://www.fda.gov/emergency-preparedness-and-response/coronavirus-disease-2019-covid-19/covid-19-vaccines

†
https://www.cdc.gov/vaccines/covid-19/health-departments/breakthrough-cases.html

§
https://www.cdc.gov/coronavirus/2019-ncov/cases-updates/variant-surveillance/variant-info.html

¶
https://covid.cdc.gov/covid-data-tracker/#vaccinations

**
https://covid.cdc.gov/covid-data-tracker/#cases_totalcases

††
https://covid.cdc.gov/covid-data-tracker/#vaccination-demographic

§§
https://www.cdc.gov/coronavirus/2019-ncov/cases-updates/variant-proportions.html

¶¶
https://www.cdc.gov/vaccines/hcp/acip-recs/vacc-specific/covid-19.html
